# The Use of Essential Oil Embedded in Polylactic Acid/Chitosan-Based Film for Mango Post-Harvest Application against Pathogenic Fungi

**DOI:** 10.3390/polym15122722

**Published:** 2023-06-18

**Authors:** Ahmad Anas Nagoor Gunny, Siew Juan Leem, Muaz Mohd Zaini Makhtar, Nor’Izzah Zainuddin, Muhammad Huzaifah Mohd Roslim, Raja Hasnida Raja Hashim, Kavita Pusphanathan, Masoom Raza Siddiqui, Mahboob Alam, Mohd Rafatullah

**Affiliations:** 1 Faculty of Chemical Engineering & Technology, Universiti Malaysia Perlis (UniMAP), Kompleks Pusat Pengajian Jejawi 3, Arau 02600, Perlis, Malaysia; siewjuanleem977@gmail.com (S.J.L.); rajahasnida92@gmail.com (R.H.R.H.); 2 Centre of Excellence for Biomass Utilization (CoEBU), Universiti Malaysia Perlis (UniMAP), Arau 02600, Perlis, Malaysia; 3 Bioprocess Technology Division, School of Industrial Technology, Universiti Sains Malaysia, Gelugor 11800, Penang, Malaysia; kavitapusphanathan@gmail.com; 4 Indah Water Konsortium, Lorong Perda Utama 13, Bukit Mertajam 14000, Penang, Malaysia; norizzahz@pop.iwk.com.my; 5 Department of Crop Science, Faculty of Agricultural and Forestry Sciences, Universiti Putra Malaysia Bintulu Campus, Bintulu 97008, Sarawak, Malaysia; muhammadhuzaifah@upm.edu.my; 6 Chemistry Department, College of Science, King Saud University, Riyadh 11451, Saudi Arabia; mrsiddiqui@ksu.edu.sa; 7 Division of Chemistry and Biotechnology, Dongguk University, 123 Dongdaero, Gyeongju-si 780714, Republic of Korea; mahboobchem@gmail.com; 8 Environmental Technology Division, School of Industrial Technology, Universiti Sains Malaysia, Gelugor 11800, Penang, Malaysia

**Keywords:** mango, essential oil, *Melaleuca alternifolia*, post-harvest disease

## Abstract

Mango has a high global demand. Fruit fungal disease causes post-harvest mango and fruit losses. Conventional chemical fungicides and plastic prevent fungal diseases but they are hazardous to humans and the environment. Direct application of essential oil for post-harvest fruit control is not a cost-effective approach. The current work offers an eco-friendly alternative to controlling the post-harvest disease of fruit using a film amalgamated with oil derived from *Melaleuca alternifolia*. Further, this research also aimed to assess the mechanical, antioxidant, and antifungal properties of the film infused with essential oil. ASTM D882 was performed to determine the tensile strength of the film. The antioxidant reaction of the film was assessed using the DPPH assay. In vitro and in vivo tests were used to evaluate the inhibitory development of the film against pathogenic fungi, by comparing the film with different levels of essential oil together with the treatment of the control and chemical fungicide. Disk diffusion was used to evaluate mycelial growth inhibition, where the film incorporated with 1.2 wt% essential oil yielded the best results. For in vivo testing of wounded mango, the disease incidence was successfully reduced. For in vivo testing of unwounded mango to which the film incorporated with essential oil was applied, although some quality parameters such as the color index were not significantly affected, weight loss was reduced, soluble solid content was increased, and firmness was increased, compared to the control. Thus, the film incorporated with essential oil (EO) from *M. alternifolia* can be an environmentally friendly alternative to the conventional approach and the direct application of essential oil to control post-harvest disease in mango.

## 1. Introduction

The total worldwide fruit trade has grown by 40% on average over the last ten years. It has significantly expanded, from 45 million tonnes to 63 million tonnes, over the last ten years. Thus, the global fruit market is increasing faster than the global population, and Asia is witnessing the greatest increase in its global fresh fruit trade. The export and import of fruit have almost doubled in Southeast Asia. This implies that the demand for fruit is increasing. Mango is a tropical fruit that is generally grown in India, Thailand, China, and Malaysia. It provides high nutritional value for humans as it contains a high volume of vitamin C. Thus, its demand is now increasing. Based on facts released by the Food and Fertilizer Technology Centre for the Asian and Pacific Region (FFTC-AP), the production of fruit in 2017 was more than 1.45 million tons, but had decreased by about 4.16% compared to 2013 [1]. The signficant loss of fruit is due to fruit fungal disease. For instance, the fungi *Colletotrichum* spp. and *Penicillium expansum* have caused post-harvest degradation of apples [2]. Pathogenic fungi are commonly found in unripe fruit. Although they remain latent during the ripening process, during a specific stage of fruit ripening and senescence the pathogenic fungus identifies the transition and switches to the destructive necrotrophic lifestyle, resulting in fruit rotting [3]. Thus, it is crucial to preserve fruit quality.

Various chemical and physical means have been proposed to maintain fruit quality. For example, the fruit can be stored in a controlled environment, where an elevated concentration of carbon dioxide and relatively lower concentration of oxygen are used. The fruit can also be preserved by adding chemical synthetic fungicide. The chemical synthetic fungicide helps to inhibit fungal growth on the plant by either causing harm to the cells or preventing fungal development. Meanwhile, in order to develop a suitable and effective control method based on this problem, notable research effort has been devoted to product evaluation, spray timing, and the improvement of statistical models for ailment forecasting. Nevertheless, the application of chemical synthetic fungicide to fruit can pose lethal risks to living organisms and the environment. Some fungicides can cause respiratory problems and irritate human skin and eyes. Furthermore, the fungicide on fruit does not easily decompose and becomes a residue in the human body [4]. The fungicide also causes the death of aquatic organisms, because it is toxic to them, when the chemical flows into the sea or rivers. Hence, chemical synthetic fungicide must be replaced with other methods.

Recently, antifungal fruit packaging has been investigated and developed to preserve fruit in a more healthy manner. Chitosan-based film has been chosen as an interesting film-forming material [5,6]. Chitosan is nature’s second most abundant polysaccharide and it has been proven to be biodegradable, non-toxic, biocompatible, and biofunctional. It is important to note that its microbial resistance is because of the ionization of amino groups; these engage with the cell membrane, which is negatively charged. This results in the discharge of subcellular microorganism constituents in addition to proteinaceous constituents [7]. Unfortunately, the disadvantage of using chitosan-based film seems to be the cost, because it is extremely costly for chitosan to be used in fruit packaging. Thus, it is suggested that chitosan should be embedded with some chemical-forming biopolymers to create edible fruit packaging [8]. In addition, chitosan materials have insufficient mechanical properties, which limits their industrial applications. Polylactic acid has been selected to mix with chitosan because of its excellent mechanical properties, which are low flammability, low moisture-regain value, low refraction index, biocompatibility, biodegradability, and high UV protection index [9,10,11,12]. Polylactic acid can be categorized as a biodegradable, thermoplastic polyester material, and is industrially produced by lactic acid polymerization from renewable sources. This means the lactic acid utilized in production of polylactic acid is usually extracted from modified corn grains.

In addition, essential oil can also be incorporated with the film produced by polylactic acid and chitosan. Essential oil (EO) is generally extracted from a plant and is often used as a natural medicine treatment. *Melaleuca alternifolia* is an evergreen tall shrub commonly grown in Australia. Essential oil from *Melaleuca alternifolia* has some excellent antibacterial, insecticidal, allelopathic, and antimicrobial properties, which makes it an excellent choice for addition to the films. Its herbal extracts also have free radical scavenging and radioprotective components [13,14]. Therefore, this study aimed to assess the chitosan–polylactic acid film containing *Melaleuca alternifolia* essential oil in terms of its antimicrobial, antioxidant, and mechanical properties for mango post-harvest application.

## 2. Materials and Methods

The essential oil was extracted from *Melaleuca alternifolia* leaves. Anhydrous sodium sulfate was used to remove traces of water from the essential oil. Chitosan powder, glacial acetic acid, glycerol, polylactic acid, chloroform, and CNC solution were used as materials to fabricate the film. Silica gel was used as a desiccant to absorb the water vapor from the film. Methanol was used as a blank solution to test the film based on antioxidant activity, while the free radical scavenging capacity of the sample material was assessed using DPPH as the test substance. In addition, gallic acid was administered as a control sample to examine the film’s antioxidant capacity. Finally, in order to evaluate the film’s antioxidant properties, a negative control was used in the form of distilled water. *Aspergillus* sp. isolated from contaminated mango in a previous study [15] was used as a test model for in vitro and in vivo antifungal activity. Potato Dextrose Agar (PDA) was used to cultivate the fungi and supply nutrients for fungal growth. Mango was used as an infection medium.

### 2.1. Extraction of Essential Oil

For the extraction of the essential oil from tea tree leaves, a microwave extraction process was used; it is important to note that the extraction process was without solvent. Five hundred grams of tea tree leaves was weighed and placed in 500 mL Erlenmeyer flasks without water or any other solvent. Ethos X- Rapid microwave, set at 500 W and 30 minutes’ extraction time, was used for this purpose. During the process, the extracted oil was collected and stored in a sample bottle. After finishing the extraction, anhydrous sodium sulfate was used to dry the essential oil followed by storage at 4 °C [16]. Later, the yield of extracted essential oil was calculated using Equation (1):

(1) $$R_{EO}\left(\%\right)=\frac{m_{EO}}{m_{S}}\times 100\%$$

where $R_{EO}$ is the yield of essential oil (%), $m_{EO}$ is the mass of essential oil in grams, and $m_{S}$ is the mass of starting material in grams. Using the above extraction process, a 0.4% yield was obtained for the EO.

### 2.2. Film Preparation

Polylactic acid and chitosan are two main ingredients that can be used to fabricate film for fruit packaging. The film was fabricated by mixing polylactic acid, chitosan, and chloroform. Firstly, 1.5% chitosan solution was produced. Polylactic acid was pre-dried in a vacuum drying oven for 3 h and then it was vacuum cooled [17]. An exactly weighed quantity of 1.2 g of chitosan powder was mixed with 80 mL of distilled water (DW) and stirred continuously with an additional 1.6 mL of glacial acetic acid solution, and, finally, 0.8 mL of glycerol was also added to the mixture. The resulting solution was stirred at 60 degrees Celsius until the chitosan powder was dissolved. Further, the polylactic acid solution was produced. Then, 1 g of polylactic acid was introduced to 30 mL of chloroform. The mixture was then stirred until the polylactic acid was dissolved; then, the CNC solution (0.3 mL) was added to the mixture. Varied essential oil concentrations were added to the mixture until it was homogenized, such as 0.3 wt%, 0.6 wt%, 0.9 wt%, and 1.2 wt% [18]. Then, 1.648 mL of 1.5% chitosan solution was added to the polylactic acid solution. The mixture was steadily stirred for a few hours until it was homogenized. The mixture was left to release bubbles at room temperature. The obtained casting solution was spread on a glass plate and chloroform was allowed to evaporate. Finally, the film was able to be peeled off.

### 2.3. Mechanical Test of the Film

#### Tensile Strength

A tensile test was performed in accordance with the ASTM (American Society for Testing and Materials) D882 standard. This method is used to determine the tensile characteristics of thin plastic sheeting. The tensile test was designed specifically for materials that are less than 1 mm thick. Firstly, the film was cut into a rectangular shape having dimensions of 10 cm × 1 cm. The rectangular strip of film was positioned in the grips of the universal testing machine, which were tightened to the appropriate degree to prevent the specimen from slipping throughout the test. The speed during the assessment was 20 mm/min and the load cell of the machine is 1 kN. The machine was started, and the film was pulled until it broke. Tensile strength was calculated using Equation (2) [19]. The others tensile parameters were obtained automatically from the computer.

(2) $$Tensile\;strength=\frac{Maximum\;load}{Original\;minimum\;cross\;sectional\;area\;of\;the\;sample}$$

where tensile strength is in N/m^2^, maximum load is in N, and cross-sectional area of the sample is in m^2^.

### 2.4. Antioxidant Activity

#### 2.4.1. Preparation of Film Extract

Before evaluating the antioxidant activity of the film, the film was first required to be extracted. This is due to the solid sample being unable to be measured using UV-VIS spectrophotometers. Hence, the film had to be extracted in a liquid form [20,21,22]. Firstly, the film that was incorporated with essential oil was cut into small pieces. Then, it was weighed to be 0.1 g. The small pieces of film were mixed with 2 mL of methanol [23]. Next, the mixture was vigorously vortexed for three minutes. The reaction mixture was allowed to stand for three hours in a dark environment in which room temperature was maintained. For three minutes, the mixture was vortexed vigorously followed by centrifuging for ten minutes at 2300 rpm. After that, the supernatant was collected and tested for DPPH scavenging activity.

#### 2.4.2. DPPH Scavenging Activity

The 2,2-diphenyl-1-picrylhydrazyl (DPPH) screening test is commonly used to assess the sample material’s free scavenging activity. Blois developed this method in 1958 to determine antioxidant reactions using a similar means using a stable free radical, -diphenyl—picrylhydrazyl (C_18_H_12_N_5_O_6_) [24]. Firstly, this experiment was conducted in a dark room as the coloring of DPPH could be seen more clearly in dark conditions. Four cuvette samples were prepared, which were the blank solution, positive control, negative control, and film sample extract [25]. For the blank solution, 3 mL of methanol was prepared. For the positive control, 2 mL of DPPH, 200 μL of gallic acid, and 800 μL of methanol were combined. This also applied for the negative control where 2 mL of DPPH, 200 μL of distilled water, and 800 μL of methanol were mixed. For the sample solution, 2 mL of DPPH, 200 μL of extract, and 800 μL of methanol were mixed. Absorbance of the sample was measured using a spectrophotometer against the blank solution at a fixed wavelength of 517 nm. Scavenging DPPH free radicals showing a drop in sample absorbance were used to monitor the film’s antioxidant activity.

DPPH radical scavenging activity was assessed based on Equation (3):

(3) $$Radical\;scavenging\;activity\;\left(\%\right)=\left[1-\left(\frac{A_{sample}}{A_{control}}\right)\right]\times 100\%$$

where $A_{sample}$ and $A_{control}$ are absorbances of the sample and control, respectively.

### 2.5. Antifungal Activity

The fungal spore suspension was spread and inoculated on a PDA medium. The film made with polylactic acid, chitosan, and essential oil was cut into a round shape. The sterilized 6 mm diameter paper discs, with distilled water, chemical fungicide, normal film, and films with different concentrations of essential oils, were placed at the center of the inoculated agar. Distilled water was used as a control. The agar plates were tightly covered with parafilm and incubated at 25 °C for seven days. Generally, the antifungal agent diffused into the agar and inhibited the growth of fungi [26]. The inhibition zone of two films under different conditions was established. Thus, the diameter of inhibition growth was noted in terms of inhibition of mycelia growth percentage (IMG, %). Using Equation (4), the percentage of inhibition of fungi mycelia growth by a test film was calculated [27]:

(4) $$Inhibition\;of\;mycelia\;growth\left(\%\right)=\frac{dc-dt}{dc}\times 100\%$$

where dc is the average diameter of the colony in the control sample and dt is the average diameter of the colony in the sample with test films.

### 2.6. In Vivo Antifungal Assay on Artificially Wounded and Inoculated Mangoes

This in vivo test was conducted using the method as stated by Campos-Requena et al. [28]. First, every mango was given a wound measuring 2 mm deep and 3 mm wide. A few of the cuts were made on the equatorial side using a sterile cork borer. Distilled water, chemical fungicide (Globus 5.5), and the film extract incorporated with essential oil were applied to the wounds. For the application of film incorporated with essential oil, two methods were used to test the effect of the film on fungal growth, which were method A and method B. In method A, the film extract was applied to the wounds on the mango and the film was placed in the container that stored the mango [29]. In method B, the film extract was only applied to the wounds on the mango [28]. Distilled water served as a control.

For two hours, both the control and doused mangoes were kept at room temperature. Then, 20 L of pathogenic fungi were spread and inoculated on each mango wound [30]. The control and treated mangoes were then stored in a labeled plastic container. For ten days, they were kept at 25% and 95% relative humidity. After ten days, rotten wounds formed on the mangoes. The number of rotten wounds on the mangoes was counted and expressed in terms of the disease incidence. The disease incidence (%) (DI) was calculated using Equation (5) [31]:

(5) $$DI\left(\%\right)=\frac{number\;of\;rotten\;wound}{number\;of\;total\;wound}\times 100\%$$

where DI is disease incidence in percentage, the number of rotten wounds of mango was counted after 10 days, and the number of total wounds is the sum of those for the control and doused mangoes.

### 2.7. In Vivo Antifungal Assay of Naturally Infected Development on Unwounded Mangoes

#### 2.7.1. Weight Loss

The mangoes were initially weighed and weights were recorded. After ten days, the mangoes were weighed again and weights were recorded. The weight loss of each mango was calculated using Equation (6):

(6) Weight loss of mango=Weight loss of mango after 10 days−Intial weight of mango

where weight loss of mango is in grams, weight of mango after 10 days is in grams, and initial weight of mango is in grams.

#### 2.7.2. Color Index

To identify the color changes of the mangoes after ten days, a colorimeter was used. A Minolta Chromameter coupled with a Minolta DP-301 data processor was used to analyze the color changes of the mangoes. Color was evaluated using the Commission Internationale de I’Eclairage (CIE) standards and expressed in three color parameters, which are L*, a*, and b* [31]. The coordinates L*, a*, and b* refer to the lightness of the color, and were used to calculate the mean values of the color index (CI) using Equation (7) [32].

(7) $$\mathrm{Colour}\;\mathrm{index}\;(\mathrm{CI})=\frac{1000\times a*}{L*\times b*}$$

where a* refers to a scale from greenness (−) to redness (+), b* refers to a scale from blueness (−) to yellowness (+), and L* refers to a scale from black (0) to white (100).

#### 2.7.3. Firmness

A texture analyzer was used to apply scientific methods to analyze the texture and firmness of the fruit. For instance, TA.XT plus C was used to measure virtually all characteristics of the fruit, such as hardness, fracturability, chewiness, adhesiveness, extensibility, and gel strength. A few parameters were used to construct the penetration texture profile obtained from Texture Expert Software, such as time, distance, and force during the analysis process. The firmness of the mangoes was recorded as the maximum positive force [15].

#### 2.7.4. Soluble Solid Content

Soluble solid content (SSC) is one of the critical fruit quality indices that influences the taste, flavor, and maturity of the fruit. For the measurement of soluble solid content (SSC), a digital refractometer was used to measure the degree to which the light changes direction, called the refractive index. Firstly, distilled water was used to calibrate the digital refractometer. A zero reading was then shown on the screen of the digital refractometer. Two drops of juice were lowered onto the prism surface until the well was completely filled. The start button of the refractometer was pressed and the result was displayed on the screen of the refractometer. Later, the surface of the prism surface was cleaned before the next measurement [15].

#### 2.7.5. Statistical Analysis

Each data set was provided as the mean and standard deviation. ANOVA (one-way; Microsoft Excel 2010) was performed with a probability level of less than 5% (*p* < 0.05). A *p* < 0.05 value indicated statistically significant findings.

## 3. Results and Discussion

### 3.1. Physical Properties of the Film

**Figure 1:**
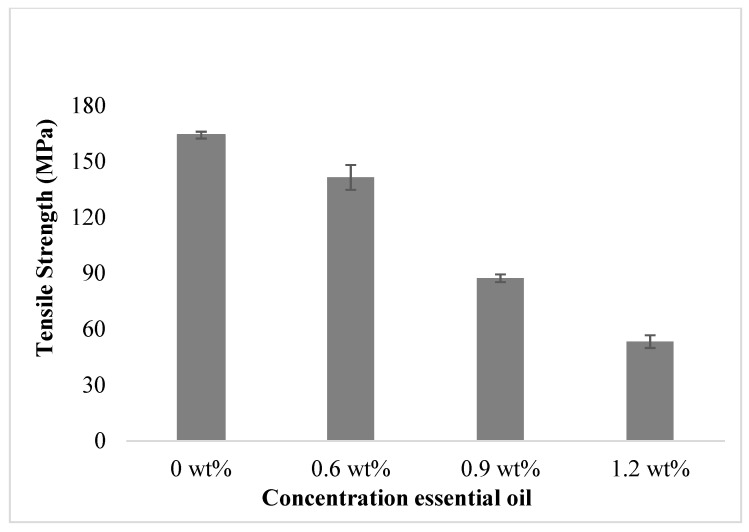
The effect of EO concentration on film tensile strength.

ASTM D882 was used to assess the strength of the film with various essential oil concentrations of 0 wt%, 0.6 wt%, 0.9 wt%, and 1.2 wt%. The overall effect of the concentration of essential oil on tensile strength is shown in Figure 1. It can be observed from the figure that the films with 0% essential oil have the highest tensile strength value, which is 164.2 ± 1.82 MPa. By comparison, the films with 1.2 wt% essential oil resulted in the lowest tensile strength value of 53.3 ± 3.40 MPa. A previous study also found that as the dosage of essential oil introduced grows, the structural rigidity of the film decreases. From the previous study, when there was an increase in cinnamon essential oil, tensile strength and break elongation of the film decreased gradually [33]. Incorporating essential oil into film reduces the molecular interaction between polymeric chains, thus resulting in materials with lower tensile strength.

**Figure 2:**
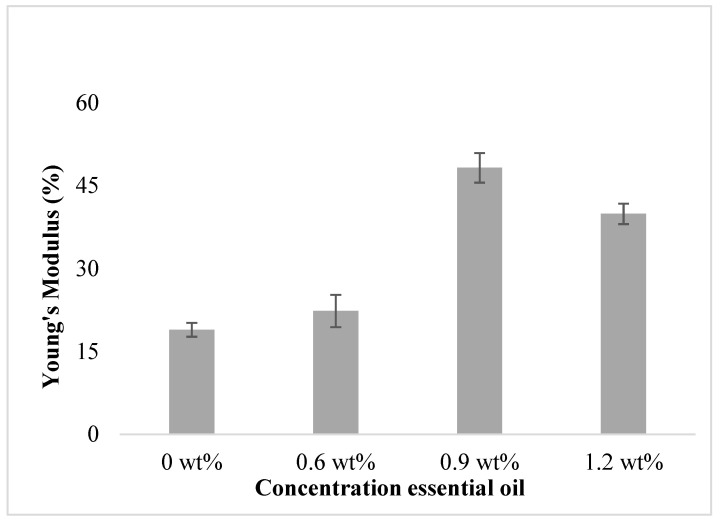
The effect of EO concentration on the film’s Young’s modulus.

The overall effect of the essential oil concentration on Young’s modulus is shown in Figure 2. As the concentration of essential oil added increases, Young’s modulus of the film increases. However, when the concentration of essential oil added is 1.2 wt%, Young’s modulus of the film slightly decreases. Although the film with 0.9 wt% essential oil exhibits the highest Young’s modulus, it has poor antioxidant and antifungal properties, compared to the film with 1.2 wt% essential oil. Somehow, the film with 1.2 wt% essential oil exhibits a higher Young’s modulus compared to the film without essential oil. This allows for the interaction of the chains, thus favoring the formation of a denser and more rigid matrix [34]. The film with 1.2 wt% essential oil will have a more rigid matrix than the film without essential oil.

**Figure 3:**
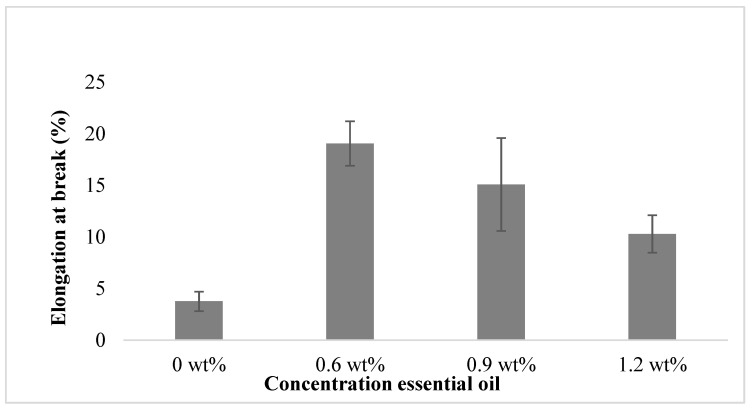
The effect of essential oil concentration on film elongation at break.

Next, the flexibility or stretching ability of the film was measured by the elongation test. The overall concentration effect of essential oil on the break elongation of the film is shown in Figure 3. The results show that the films embedded with essential oil are less brittle than the film without essential oil, and the latter showed a lower elongation. In general, these tests showed that adding essential oil to the film reduced tensile strength and enhanced the flexibility of the film. These observations are probably because the hydroxyl group on the EO chain caused the formation of hydrogen bonds, which replace the internal hydrogen bonds between polymers; these lead to increasing the free spaces between molecules, hence reducing stiffness and increasing film flexibility [35]. However, when the concentration of essential oil was further increased to more than 9 wt%, the elongation at break was reduced. Similar observations were reported for PLA-based formulations with active ingredients such as *Origanum vulagare* L. [36].

### 3.2. DPPH Antioxidant Test

**Figure 4:**
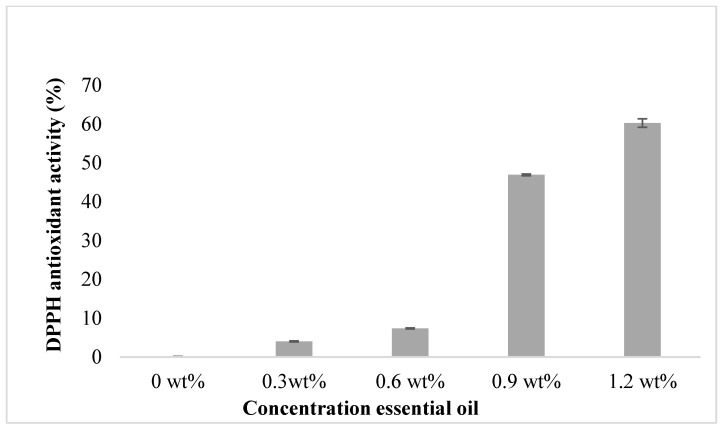
Graph of DPPH scavenging activity (%) versus the concentration of essential oil.

In terms of antioxidant properties, the DPPH radical scavenging reaction has been used to assess the films’ antioxidant activity [37,38]. As shown in Figure 4, the introduction of EOs to films improved their antioxidant characteristics in comparison to the film without essential oil. The film without essential oil showed scavenging activity of only 0.1206 ± 0.03%. The scavenging activity increased as the concentration of the essential oil in the film increased. The highest antioxidant activity was 60.2653 ± 1.11% for the 1.2 wt% essential oil. This observation is in line with a previous study by Carmen Ballester-Costa and team where they found the radical scavenging reaction of chitosan films significantly increased as the concentration of essential oil increased [39]. The chitosan film without essential oil only showed a slight scavenging activity on DPPH. Furthermore, it has been shown that the DPPH free scavenging activity increased with the increasing concentration of tea tree oil. Three terpenic compounds found in the essential oil from tea tree, *Melaleuca alternifolia*, which are α-terpinene, α-terpinolene, and γ-terpinene, contributed to the major antioxidant activity in the oil [40]. Hence, this shows that essential oil embedded in the film plays a major role in improving the scavenging activity of the film.

### 3.3. In Vitro Antifungal Test

An in vitro antifungal test was performed to evaluate the essential-oil-encapsulated film’s ability to combat the selected fungi. From Table 1, the highest in vitro activity of 72.3 ± 2.01% was recorded for chemical fungicide (Globus 5.5), followed by the film embedded with 1.2 wt% essential oil from *Melaleuca alternifolia*, for which 45.5 ± 2.49% was recorded. In contrast, the film without essential oil did not show any inhibition effects. These observations show that incorporating essential oil into the film improved antifungal activity compared to the film without any essential oil. This is probably due to the presence of compounds such as terpinen-4-ol and α-terpineol in the essential oil from tea tree, *Melaleuca alternifolia*, which exhibits strong antimicrobial properties against fungi [41]. The exhibited antimicrobial actions could be explained by the synergy between these compounds and the cell membrane of the target microorganisms [42].

### 3.4. In Vivo Antifungal Test

**Figure 5:**
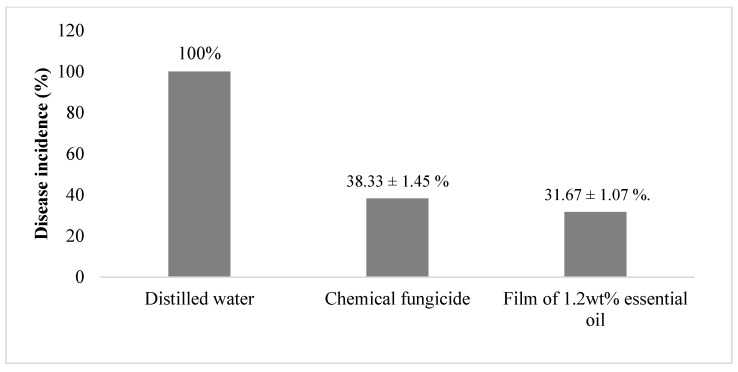
The impact of various treatments on disease incidence.

**Figure 6:**
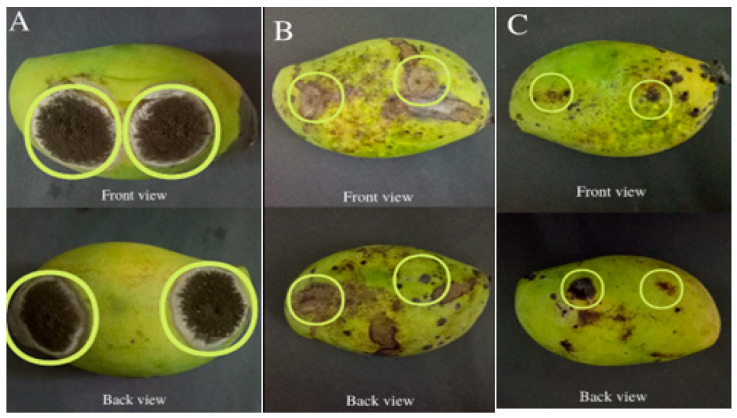
Appearance of fungal growth at the wound area of inoculated mangoes after 10 days: (**A**) deionized water as a control, (**B**) a fungicide (Globus 5.5), and (**C**) 1.2 wt% essential oil film.

Figure 5 and Figure 6 show that mango with distilled water had no disease incidence. Mango treated with a chemical fungicide had a disease incidence of 38.33 1.45%, whereas mango treated with a film containing 1.2 wt% essential oil had a disease incidence of 31.67 ± 1.07% on artificially inoculated mango. The application of both chemical fungicide and film with 1.2 wt% essential oil affected the antifungal activity on the mangoes, where these methods minimized the formation of mycelium and sporulation in comparison to the control sample (distilled water). For the application of distilled water on inoculated mango, it was shown that there was black mold decomposition on the wound. Meanwhile, for the application of chemical fungicide, it was shown that there was only the growth of soft water-soaked lesions around the wound. For the application of film with 1.2 wt% essential oil, it was shown that only mold grew on the mango.

### 3.5. Quality Assessment of Post-Harvest Mango

Post-harvest mango quality between control and treatment groups was assessed by comparing the color, soluble solid content (SSC), weight loss, and firmness properties between treated and untreated post-harvest mangoes. As shown in Table 2, there is an effect of distilled water, chemical fungicide, and film with 1.2 wt% EO on weight loss and firmness of the mangoes. The data revealed that film embedded with 1.2% essential oil reduced the weight loss of the post-harvest mango compared to chemical fungicide treatment and the control. The film treatment showed the least weight loss. The high weight loss in mango when compared to the control (distilled water) is due to water loss from transpiration and respiration mechanisms. By applying essential oil film to the mango, the coating of film on the external surface serves as a buffer to moisture loss, thereby retaining much of the mango’s moisture content and thus reducing weight loss. The hydrophobic components of essential oil in the coatings contribute to the weight loss reduction. The hydrophobic properties of essential oil aid in the formation of a continuous phase around the fruit, reducing moisture loss from the fruit by forming a water barrier on the fruit’s surface [43]. In addition, a study conducted by Chafer et al. [44] found that the chitosan coatings incorporated with tea tree oil showed a reduced weight loss of the fruit compared to the control. Hence, post-harvest mango control using film embedded with essential oil will increase the selling price, as consumers generally do not prefer light fruit [45].

Next, the film treatment showed the highest firmness. Mango fruit ripening is characterized by a loss of firmness caused by the cell wall’s digestive processes carried out by pectin esterase, polygalacturonase, and other enzymes [46]. Changes in fruit firmness are mainly due to water loss in fruit. The high firmness of the essential-oil-coated mango film could be related to the coating’s permeability and its effect on the fruit. As a result, the evaporation of water can be reduced and shrinkage of mango can be avoided. In a previous study, the chitosan coatings incorporated with tea tree oil showed a higher firmness of the fruit compared to the control [43]. Thus, the mango to which film with 1.2 wt% EO was applied showed the highest firmness. For soluble solid content (SSC), the mango to which the film with essential oil was applied recorded the highest value. The high value of SSC in the mango coated with the film is because the protective oxygen barrier reduced the oxygen supply on the mango surface, thereby inhibiting respiration. The low SSC value in mango to which distilled water was applied was caused by a decrease in the number of carbohydrates and pectins, partial hydrolysis of protein, and glycoside decomposition into sub-units during respiration [47]. However, there was no significant effect of distilled water, chemical fungicide, or film with 1.2 wt% essential oil on the color index of the mangoes. A previous study found there was no substantial difference in the color index of the minimally processed mango to which chitosan coatings with citric essential oil were applied [48].

## 4. Conclusions

Chitosan–polylactic acid films containing *Melaleuca alternifolia* essential oil (EO) were successfully produced and showed active packaging functions to control post-harvest diseases in mango. The incorporation of *Melaleuca alternifolia* essential oil into the film also improved the flexibility and elongation at break of the film. The DPPH scavenging activity was also found to be directly proportional to the essential oil’s concentration in the film, as the increase in the concentration of essential oil caused an increased in the DPPH scavenging activity. Meanwhile, the film containing 1.2 wt% EO performed well in in vitro and in vivo antifungal testing, preventing the spread of the fungus responsible for post-harvest mango diseases. The application of the film with 1.2 wt% essential oil to mango exhibited an improvement in and positive effect on the fruit quality. It showed a reduction in weight (g) loss and an increase in firmness (g/f). At the same time, it did not exhibit a significant effect on the color index or soluble solid content (SSC) compared to other samples. Given that the film integrated with EO from *Melaleuca alternifolia* is comparable to a chemical fungicide, it can be a superior alternative for preventing mango post-harvest disease. As a result, it is a more effective and affordable solution to managing mango’s pathogenic diseases. Moreover, the use of film incorporated with essential oil is very economical compared with the direct application of essential oil on the crops. Hence, to produce chemical-free fruit and vegetables, and protect the environment, consumer, and farmer, the development and improvement of film incorporated with essential oil should be a focus and concern.

## Figures and Tables

**Table 1:** Effect of control, chemical fungicide, and film with 1.2 wt% essential oil from *Melaleuca alternifolia* on mycelia growth.

Treatments	Mycelia Growth (mm)	Mycelia Growth Inhibition (%)
Deionized water as control	-	-
Fungicide (Globus 5.5)	21.7 ± 1.53	72.3 ± 2.01
Normal film	6.0	-
Film + 1.2 wt% essential oil	11.0 ± 0.50	45.5 ± 2.49

**Table 2:** Effect of control (distilled water), chemical fungicide, and film of 1.2 wt% essential oil from *Melaleuca alternifolia* on weight loss (%), color index, SSC (%), and firmness (g/f).

Treatment	Colour Index	SSC (%)	Percentage of Weight Loss (%)	Firmness (g/f)
Control (distilled water)	−3.38 ± 1.17	11.93 ± 0.59	13.83 ± 0.31	655.0 ± 7.76
Chemical fungicide	−2.62 ± 1.17	12.20 ± 0.96	10.59 ± 0.09	792.77 ± 36.79
The film with 1.2wt% essential oil	−4.14 ± 1.09	13.93 ± 0.50	9.55 ± 0.08	867.83 ± 23.53

## Data Availability

The data that support the findings of this study are available from the corresponding authors upon reasonable request.
